# Research on Tunnel Crack Identification Localization and Segmentation Method Based on Improved YOLOX and UNETR++

**DOI:** 10.3390/s25113417

**Published:** 2025-05-29

**Authors:** Wei Sun, Xiaohu Liu, Zhiyong Lei

**Affiliations:** 1 School of Mechatronic Engineering, Xi’an Technological University, Xi’an 710021, China; 2 School of Mechanical Engineering, Shaanxi University of Technology, Hanzhong 723001, China; 3 Trine Engineering Institute, Shaanxi University of Technology, Hanzhong 723001, China; lxh@snut.edu.cn; 4 School of Electronic Information Engineering, Xi’an Technological University, Xi’an 710021, China; gemlei@xatu.edu.cn

**Keywords:** tunnel cracks, crack identification, crack segmentation, lightweight network, attention mechanism

## Abstract

To address the challenges in identifying and segmenting fine irregular cracks in tunnels, this paper proposes a new crack identification, localization and segmentation method based on improved YOLOX and UNETR++. The improved YOLOX recognition algorithm builds upon the original YOLOX network architecture. It replaces the original CSPDarknet backbone with EfficientNet to enhance multi-scale feature extraction while preserving fine texture characteristics of tunnel cracks. By integrating a lightweight ECA module, the proposed method significantly improves sensitivity to subtle crack features, enabling high-precision identification and localization of fine irregular cracks. The UNETR++ segmentation network is adopted to realize efficient and accurate segmentation of fine irregular cracks in tunnels through global feature capture capability and a multi-scale feature fusion mechanism. The experimental results demonstrate that the proposed method achieves integrated processing of crack identification, localization and segmentation, especially for fine and irregular cracks identification and segmentation.

## 1. Introduction

Tunnel cracks represent a significant hidden risk to the safe operation of road tunnels, as their progression can lead to the instability of lining structures, exacerbate water seepage and leakage and even trigger collapse incidents. Crack identification is an indispensable component for ensuring tunnel structural safety and stability. Its importance is self-evident, especially for fine and irregular cracks, which directly reflect structural health conditions and exhibit characteristics such as concealment, minuteness and rapid development. If left undetected and untreated, these fine irregular cracks may lead to severe safety hazards over time, potentially resulting in catastrophic tunnel failures. Regular tunnel crack identification, timely detection and repair play crucial roles in preventing structural damage, ensuring transportation safety, while the research and development of efficient and accurate fine irregular crack identification technologies hold significant importance for guaranteeing tunnel operational safety and extending service life.

In recent years, tunnel crack detection has primarily relied on static image acquisition methods. Most recognition methods depend on specific features such as shape, texture or intensity variations, while fine irregular cracks often lack distinctive feature signatures, which leads to reduced recognition accuracy. Furthermore, conventional approaches mainly rely on human expertise and visual inspection, which not only consume significant time and effort with low efficiency, but also struggle to ensure the accuracy and consistency of recognition results. These methods, which demonstrate limited capability in handling noise or complex backgrounds, are susceptible to environmental interference, and typically require large amounts of annotated training data, whereas fine irregular crack recognition often suffers from scarce samples and substantial training difficulties.

Researchers have conducted extensive studies on tunnel crack recognition and segmentation using deep learning approaches to address the challenging identification problem. Bao et al. [1] proposed the M-YOLO algorithm that achieves accurate crack recognition through innovative techniques, effectively solving multiple challenges. Huang et al. [2] employed U-net, VGG-16 and Faster R-CNN combined with image restoration technology to realize high-accuracy crack recognition. Zhou et al. [3] integrated self-attention mechanisms with CNN and adopted dual-branch feature extraction to improve crack recognition accuracy. Song et al. [4] established the first dataset with semantic segmentation annotations and proposed a fast recognition algorithm that significantly improved identification efficiency. Additionally, Wu et al. [5] combined improved Retinex with deep learning to efficiently segment crack images through image enhancement and VGG19 network, obtaining precise crack dimension information that provides reliable basis for tunnel health assessment. Song et al. [6] developed the Mobile-PSPNet method that achieved real-time segmentation of tunnel lining cracks while improving computational efficiency. Kuang et al. [7] introduced a lightweight Transformer architecture that utilizes self-attention mechanisms to capture long-range dependencies of cracks, enhancing segmentation accuracy. Chen Y. et al. [8] improved crack segmentation accuracy and robustness by combining morphological networks with multiple loss mechanisms. Wu et al. [9] proposed PCTNet, a Transformer-based semantic segmentation network for detecting pixel-level crack width, demonstrating good generalization and robustness in complex environments. Although these methods achieve high recognition rates for obvious tunnel cracks, existing approaches [10,11,12,13,14] have inherent limitations when addressing, multi-scale feature loss causing resolution constraints in imaging fine irregular cracks, where micro-textures and macro-structural features exhibit cross-scale coupling. The fixed receptive field mechanism of traditional CNNs [15,16] struggles to simultaneously capture local crack details and global contextual relationships, resulting in high-frequency information loss during feature pyramid fusion. Furthermore, high noise background interference, including prevalent water stains, repair marks and lighting reflections on tunnel walls, exhibits high grayscale and texture similarity to actual cracks, making tunnel crack identification particularly difficult. These issues result in inadequate performance for fine irregular crack detection, often missing fine variations and producing inaccurate recognition results.

Although current deep learning methods have made progress in conventional crack detection, they still show limitations in detecting fine irregular cracks and often fail to retain subtle fine irregular crack texture features during recognition. This paper proposes an improved YOLOX and UNETR++-based method for crack identification, localization and segmentation. The contributions of this paper are as follows.

(1) We propose the improved YOLOX algorithm for crack identification and localization, incorporating EfficientNet as the backbone network to enhance the model’s feature extraction capability. EfficientNet employs efficient convolution and compound scaling to enhance detail sensitivity with low computational overhead, effectively capturing fine irregular crack features. To further optimize performance, we integrate the lightweight ECA mechanism following the EfficientNet module, forming the EfficientNet–ECA module. This combination enhances the model’s sensitivity to fine, irregular cracks without introducing additional computational or memory burdens, thereby improving detection reliability in complex tunnel environments.

(2) Based on the accurate identification and location of tunnel cracks, we propose the UNETR++ algorithm to segment the tunnel crack image and set up the segmentation network model. By utilizing the UNETR++ algorithm to segment tunnel cracks, the specific shape and distribution of cracks can be captured in more detail. This approach not only enhances the efficiency of segmenting fine irregular tunnel cracks but also ensures high accuracy in the segmentation process.

This paper is structured as follows: Section 2 describes the overall design of crack identification, localization and segmentation. Section 3 tunnels a detailed expansion of the crack identification, localization and segmentation method based on improved YOLOX and UNETR++. Section 4 gives a detailed description of the experimental preparations for the training of the crack identification model, and Section 5 carries out the experimental analysis. Finally, the conclusions are drawn in Section 6.

## 2. The Overall Design for Crack Detection–Localization and Segmentation

**Figure 1:**
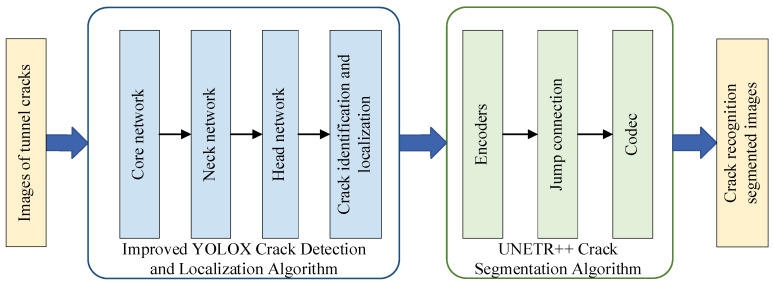
The overall design flowchart for crack identification, localization and segmentation.

This paper develops a tunnel fine irregular crack recognition algorithm that integrates crack identification, localization and segmentation functions. The algorithm effectively combines the improved YOLOX network model with the UNETR++ segmentation network, achieving complete processing from initial detection to precise segmentation of tunnel cracks. Figure 1 illustrates the overall architecture of the crack identification, localization and segmentation system.

The drone-collected tunnel crack images are first processed by the improved YOLOX crack recognition algorithm, which inputs the images into the backbone network. The backbone network serves as the core for feature extraction, replacing the original YOLOX Focus structure with convolutional down-sampling and incorporating the EfficientNet-ECA module for feature extraction to obtain key image features. The neck network performs feature fusion to further enhance the model’s ability to extract discriminative features. The improved YOLOX adopts a decoupled head mechanism at the output layer, enabling more flexible processing of multi-scale semantic information and demonstrating strong scalability to accommodate various task requirements. Compared with traditional crack detection methods, the improved YOLOX model more accurately captures fine, irregular cracks in tunnel images, providing reliable localization information for subsequent crack segmentation. The recognized and localized images are then fed into the UNETR++ segmentation network. Using deep learning techniques, UNETR++ automatically learns and extracts essential features from the crack images, and through the integration of the EPA module, it achieves precise segmentation of crack regions.

The improved YOLOX network model combines with the UNETR++ image segmentation network to construct an efficient and accurate tunnel crack recognition algorithm. This algorithm not only rapidly and accurately identifies crack locations in complex tunnel environments but also performs precise segmentation of crack regions, providing strong support for subsequent analysis and processing.

## 3. Tunnel Crack Identification Localization and Segmentation Based on Improved YOLOX and UNETR++

### 3.1. YOLOX Algorithm

**Figure 2:**
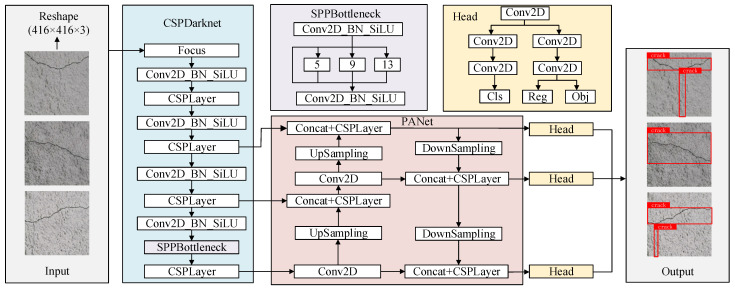
YOLOX network structure.

The YOLOX [17,18] algorithm implements refined improvements and sophisticated combinations in decoupled heads, anchor boxes and data augmentation, achieving comprehensive accuracy advantages over YOLOv3, YOLOv4 and YOLOv5 [19,20,21]. The YOLOX tunnel lining crack detection algorithm consists of a CSPDarknet backbone network, PANet feature extraction network and Head output module, with its algorithmic principle shown in Figure 2.

The input images are first preprocessed through standardization and uniformly resized to a fixed dimension of 416 × 416. They are then fed into the backbone feature extraction network, where multi-layer convolutional operations extract three effective feature matrices at different scales with dimensions of 52 × 52 × 256, 26 × 26 × 512 and 13 × 13 × 1024. These feature matrices correspond to feature information under different receptive fields. The three feature layers are subsequently input into an enhanced feature extraction network, where meticulously designed operations including convolution, up-sampling, down-sampling and feature fusion strengthen and integrate the original features, capturing richer and more detailed image features, ultimately outputting three enhanced feature layers. The enhanced feature layers are then transmitted to the prediction module, where each layer undergoes one convolutional operation before being split into two parallel processing paths for classification and regression. The classification path performs two 3 × 3 convolutions and one 1 × 1 convolution to generate predictions of object categories corresponding to feature points. The regression branch performs two 3 × 3 convolutions, with one sub-branch using 1 × 1 convolution to adjust the bounding box position and the other using 1 × 1 convolution to determine the validity of the detected objects. Finally, the classification and regression prediction results from each feature layer are integrated and stacked to form complete predictive outputs, achieving object category determination and precise bounding box position prediction for each feature point in the input image.

### 3.2. Improved YOLOX Crack Identification and Localization Algorithm

**Figure 3:**
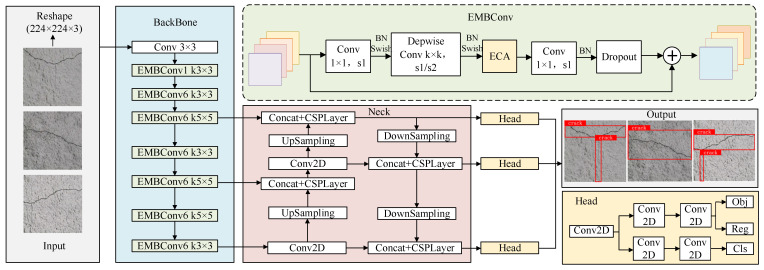
Improved YOLOX network structure.

This paper proposes an improved YOLOX algorithm for tunnel crack identification and localization to achieve efficient and precise identification of fine irregular cracks in tunnels. The algorithm modifies the YOLOX backbone network by incorporating the EfficientNet–ECA [22] module to achieve accurate crack localization. This module employs an efficient channel attention mechanism to maintain feature extraction capability while significantly reducing model parameters and computational complexity. It consequently improves sensitivity to fine irregular crack features and recognition accuracy while enhancing the model’s generalization capability. Figure 3 illustrates the architecture of the improved YOLOX network. The modified design preserves the advantages of the original detection framework while achieving dual improvements in computational efficiency and recognition performance through modular architecture. This provides a more efficient and accurate technical solution for tunnel crack detection.

In the improved YOLOX network, the system enhances feature recognition capability for fine and irregular cracks. The research replaces the CSPDarknet backbone in YOLOX with EfficientNet [23] to improve feature extraction efficiency and quality. The architecture integrates an ECA [24] (Efficient Channel Attention) module at the EfficientNet backend. This integration enables the enhanced model to more effectively capture tunnel crack features, particularly excelling at the efficient extraction of fine and irregular crack characteristics.

**Figure 4:**
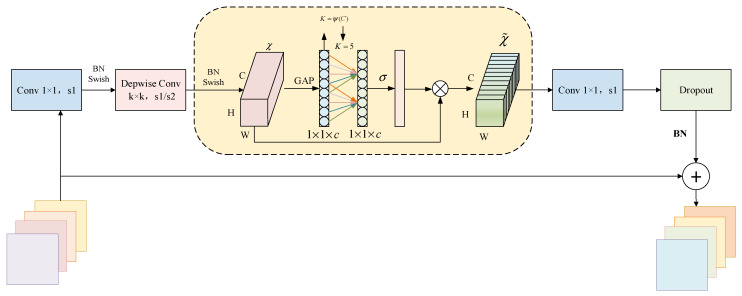
EfficientNet–ECA module.

The designed EfficientNet–ECA module integrates the ECA attention mechanism into critical feature extraction stages of the EfficientNet backbone network. This design utilizes deep feature maps that contain more abstract semantic information, while the ECA mechanism improves crack-related feature detection by emphasizing relevant features and suppressing background noise through channel weight adjustment. Figure 4 illustrates the detailed architecture of the EfficientNet–ECA module [25,26]. The module consists of a dimension-increasing Conv-1 × 1 layer, a k × k depthwise convolution layer $\left(k\in \left\{3\times 3,5\times 5\right\}\right)$, the ECA attention module, a dimension-reducing Conv-1 × 1 layer, and a dropout layer. Batch normalization (BN) is applied during training to accelerate model convergence, and the Swish activation function introduces nonlinearity, helping to mitigate overfitting.

EfficientNet is a neural architecture search-derived network structure that demonstrates superior efficiency and accuracy. It enhances feature extraction capability without significantly increasing computational requirements. The architecture incorporates an inherently efficient design, utilizing a compound scaling method with a coefficient of ϕ, which allows for flexible adjustment of network depth, width and resolution based on varying computational constraints. This approach enables the acquisition of more comprehensive and representative features with relatively low computational cost [27,28].

(1) $$d=\alpha^{\phi },$$

(2) $$w=\beta^{\phi },$$

(3) $$r=\gamma^{\phi },$$

where $\alpha ,\beta^{2},\gamma^{2}\approx 2$ (α≥1,β≥1,γ≥1), ϕ is the scaling factor.

EfficientNet employs compound scaling to enhance multi-scale feature extraction capabilities from input images. This approach enables the model to better capture critical feature information when processing diverse targets across various scenarios, consequently improving recognition accuracy. The network parameters are listed in Table 1.

The EfficientNet–ECA module replaces the original Squeeze-and-Excitation (SE) mechanism with ECA to effectively capture channel dependencies and enable cross-channel interaction. Specifically, the fully connected (FC) layer following the global average pooling (GAP) layer is replaced with a Conv-1 × 1 layer. Additionally, the kernel size is adaptively adjusted through a nonlinear mapping function ψ(C), which enhances cross-channel interactions across different ranges. For a given output channel dimension C, the kernel size K is determined by

(4) $$K=\psi (C)={\left|\frac{\log_{2}(C)}{\gamma }+\frac{b}{\gamma }\right|}_{odd},$$

where γ is set to 2 and b is set to 1. ${\left|\cdot \right|}_{odd}$ denotes taking the absolute value and rounding down to the nearest odd number to ensure an odd kernel size. Evidently, in the nonlinear mapping *ψ*, high-dimensional channels exhibit long-range interactions while low-dimensional channels demonstrate short-range interactions.

**Figure 5:**
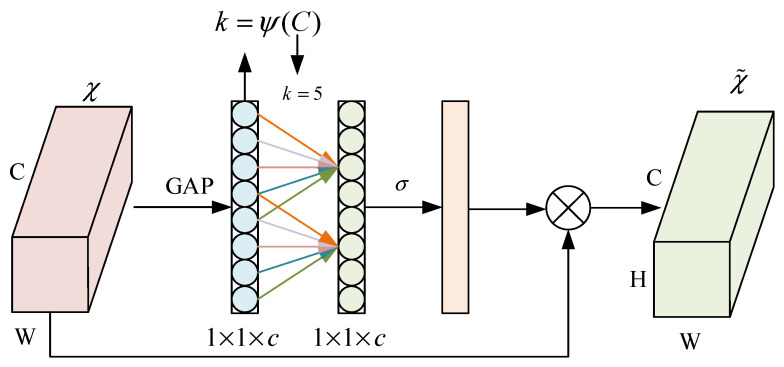
ECA attention mechanism.

The ECA attention mechanism strengthens channel features in input feature maps through its efficiency and adaptive characteristics. It directs the model’s focus towards feature channels relevant to small targets, thereby enhancing the network’s feature representation capacity. By eliminating dimensionality reduction and employing local cross-channel interaction strategies, the mechanism effectively improves model performance while reducing the number of parameters. The ECA enhances feature representation by globally adapting the weighting of each channel’s feature maps. It utilizes 1D convolution to capture inter-channel dependencies, with its architecture illustrated in Figure 5. It reduces computational complexity by learning channel attention through convolutional layers, providing deeper convolutional neural networks with stronger feature representation capabilities. This approach enables models to better focus on small target features, consequently improving small target recognition performance. Compared with conventional attention mechanisms, the ECA module achieves efficient and lightweight properties by avoiding complex dimensionality reduction and expansion processes.

As shown in the figure, given the input feature tensor $F\in R^{C\;\times \;H\;\times \;W}$, the ECA module obtains features for each channel through global average pooling (GAP) as

(5) $$GAP(F)=\frac{1}{H\times W}\sum_{i=1}^{H}\sum_{j=1}^{W}F_{c,i,j},$$

The system performs local channel interaction using 1D convolution to obtain:

(6) z=Conv1D(GAP(F)),

The Sigmoid activation function generates channel weights ω:

(7) ω=σ(z),

The importance of each channel is quantified by generating a vector of channel weights, a simple and efficient process that ensures a lightweight and high-performance model.

In this paper, the combined loss function is used to replace the original loss function. By combining CE Loss and Dice Loss, the loss of category level and pixel level can be considered comprehensively to improve the performance of the model.

Cross entropy loss (CELoss) is a loss function based on category level, which is used to measure the difference between the output probability distribution of the model and the real probability. The CE loss function is shown as follows:

(8) $$Loss_{CE}=-\sum_{i}y_{i}\log({\hat{y}}_{i}),$$

where $y_{i}$ is the *i*-th element of the true probability distribution, and ${\hat{y}}_{i}$ is the probability of the *i*-th category of the model output.

Dice Loss is a loss function based on pixel level, which is used to measure the overlap between the predicted segmentation result and the real label, and can deal with the imbalance between positive and negative samples. The Dice loss function is shown as follows:

(9) $$Loss_{Dice}=1-\frac{2\sum_{i}^{N}y_{i}{\hat{y}}_{i}}{\sum_{i}^{N}y_{i}\sum_{i}^{N}{\hat{y}}_{i}},$$

where $y_{i}$ and ${\hat{y}}_{i}$ represent the true value and predicted value of pixel *i*, respectively. *N* is the total number of pixels, which is equal to the number of pixels in a single image multiplied by batch size.

In the identification of tunnel cracks, the combination of CELoss and DiceLoss can significantly improve the recognition ability of the model in the case that the cracks may be used as small targets or have high similarity with the background. By optimizing the overlap between the predicted results and the real labels, DiceLoss makes the model pay more attention to the target features and reduce the background interference. CELoss provides effective supervision of classification tasks. The combination of the two can balance the positive and negative samples, especially in the case of few crack targets and large background areas, the positive sample information can be used more effectively to improve the recognition accuracy. This combination enables the model to learn data features from multiple dimensions, enhance robustness and generalization ability, and quickly guide the model to learn category differences, refine target area segmentation, make the training process more stable, accelerate convergence speed and improve training efficiency.

Therefore, in this paper, the combined loss function based on CE loss function and supplemented by Dice loss function is used to deal with the problem of positive and negative sample imbalance of crack image, and the weight coefficient is introduced to adjust the proportion of Dice loss function. The combined loss function is

(10) $$Loss=Loss_{CE}+\alpha Loss_{Dice},$$

where Loss is the combined loss function used in this paper; $L{\mathrm{oss}}_{CE}$ is the CE loss function; $L{\mathrm{oss}}_{Dice}$ is the Dice loss function; α(0<α<1) is the weight coefficient of the proportion of Dice loss function.

### 3.3. U-Net Segmentation Algorithm

**Figure 6:**
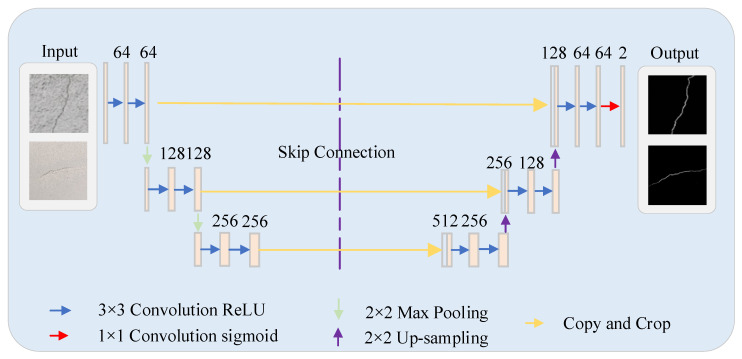
U-Net network architecture.

The U-Net [29] model represents a classical semantic segmentation architecture. Researchers originally designed it for medical image segmentation tasks. Its efficiency and flexibility have enabled widespread applications across multiple domains. Figure 6 illustrates its network architecture.

The U-Net architecture employs a U-shaped encoder–decoder structure as its core framework [30]. The encoder progressively extracts image features through convolutional and pooling layers, reducing spatial resolution while acquiring deeper semantic information. The decoder restores image dimensions via up-sampling operations and generates segmentation results by incorporating features from the encoder. Deeper network layers output feature maps containing abstract semantic features with larger receptive fields, while shallow network layers produce feature maps with detailed characteristics like texture and location. Therefore, the U-Net network structure incorporates jump connections to splice and fuse high-level features containing image details and low-level features with contextual information, retaining the spatial detail information of the image, and ultimately realizing more accurate target segmentation results.

### 3.4. UNETR++ Crack Segmentation Algorithm

**Figure 7:**
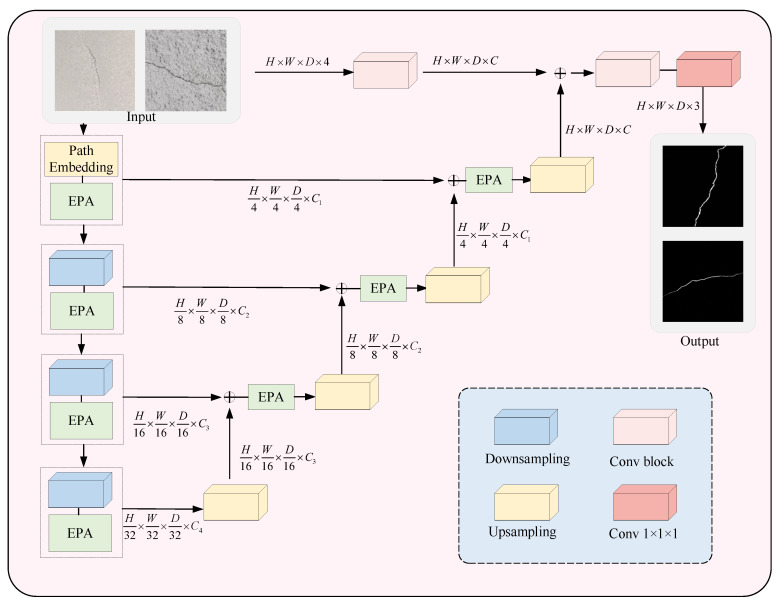
UNETR++ segmentation algorithm architecture.

To achieve more accurate crack contours and detailed features, this paper uses UNETR++ [31] for tunnel crack image segmentation following the improved YOLOX for crack localization. UNETR++ provides high-quality crack segmentation masks, ensuring efficiency in terms of parameter count, computational cost and inference speed. The key innovation is an Efficient Pairwise Attention block which employs two interdependent branches, spatial attention and channel attention. This design allows the model to effectively learn and extract discriminative features by integrating spatial and channel information. UNETR++ shares weights for the query key mapping functions between the spatial and channel branches, providing complementary benefits while significantly reducing model complexity. Figure 7 illustrates the hierarchical encoder–decoder architecture of UNETR++.

The UNETR++ framework builds upon UNETR by introducing optimizations and innovations. It incorporates distinctive skip connections between the encoder and decoder, followed by convolutional blocks to generate precise prediction masks. Unlike UNETR, which maintains a fixed feature resolution in the encoder, UNETR++ adopts a more flexible hierarchical design. This design progressively halves the feature resolution at each stage, enabling more efficient information processing and feature extraction [32].

**Figure 8:**
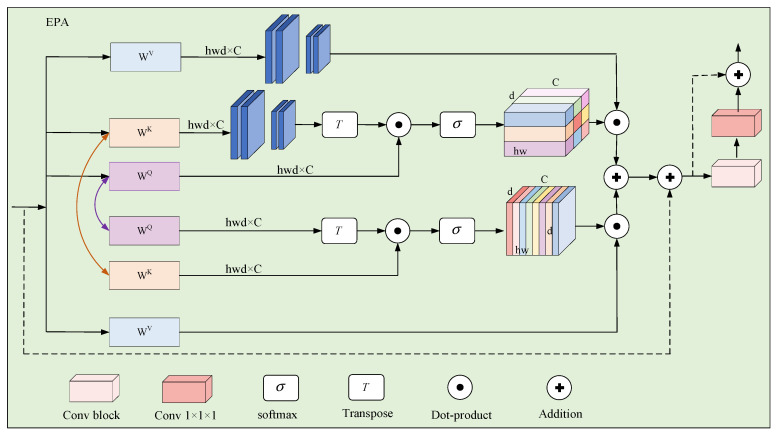
Efficient Paired Attention (EPA) block.

The encoder of UNETR++ consists of four core stages, each with the number of channels set to $\left[C1,\;C2,\;C3,\;C4\right]$. In the first stage, a patch embedding operation is applied, where the input 3D volumetric data are partitioned into a series of non-overlapping 3D patches. Each patch is then projected onto the C-channel dimension to generate a feature map. For the subsequent three stages of the encoder, UNETR++ utilizes a down-sampling layer that applies non-overlapping convolutions, effectively halving the resolution of the feature map at each stage. Each stage incorporates Efficient Paired Attention (EPA) blocks, which facilitate the efficient learning of discriminative features by employing interdependent branches of spatial and channel-wise attention mechanisms. As illustrated in Figure 8, this hierarchical structure, combined with the integration of EPA blocks, enables UNETR++ to achieve superior performance and computational efficiency in the tunnel crack segmentation task.

As illustrated in Figure 8, the input feature maps x are fed into the channel and spatial attention modules of the EPA block. The weights of *Q* and *K* linear layers are shared across the two attention modules and a different *V* layer is used for each attention module. The two attention modules are computed as

(11) $${\hat{X}}_{S}=SA(Q_{S},K_{S},V_{S}),$$

(12) $${\hat{X}}_{C}=CA(Q_{S},K_{S},V_{C}),$$

where ${\hat{X}}_{S}$ and ${\hat{X}}_{C}$ denote the spatial and channels attention maps, respectively. *SA* is the spatial attention mod ule, and *CA* is the channel attention module. $Q_{S}$, $K_{S}$, $V_{S}$ and $V_{C}$ are the matrices for shared queries, shared keys, spatial value layer and channel value layer, respectively [33].

We perform sum fusion and transform the outputs from the two attention modules by convolution blocks to obtain enriched feature representations. The final output $\hat{X}$ of the EPA block is obtained as

(13) $$\hat{X}={\mathrm{Conv}}_{1}({\mathrm{Conv}}_{3}({\hat{X}}_{s}+{\hat{X}}_{c})),$$

where ${\hat{X}}_{s}$ and ${\hat{X}}_{c}$ denotes the spatial and channels attention maps, and Conv1 and Conv3 are 1 × 1 × 1 and 3 × 3 × 3 convolution blocks, respectively.

UNETR++ enhances crack segmentation performance by integrating EPA blocks with a hierarchical encoder–decoder architecture. This combination not only improves segmentation accuracy by effectively capturing multi-scale and contextual information but also enhances the real-time applicability of the model. The hierarchical design facilitates the extraction of both global and local features through multiple levels of encoding and decoding, while the EPA blocks optimize attention mechanisms across various scales, ensuring more precise identification of fine irregular crack structures [34]. As a result, UNETR++ strikes an optimal balance between high segmentation precision and efficient real-time processing, making it particularly suitable for practical applications in tunnel crack detection and monitoring systems.

## 4. Crack Recognition Model Training

### 4.1. Tunnel Crack Dataset

**Figure 9:**
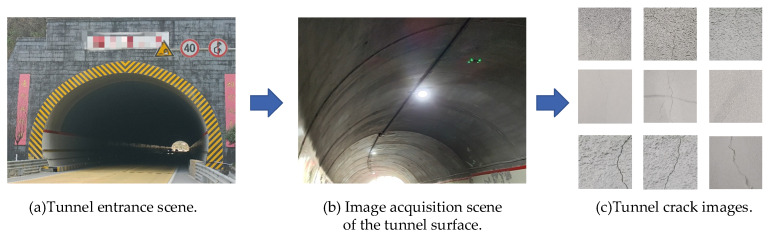
The tunnel scene and tunnel crack image.

Tunnel crack image dataset was acquired through UAV field photography of a specific tunnel, the tunnel scenario shown in Figure 9.

**Figure 10:**
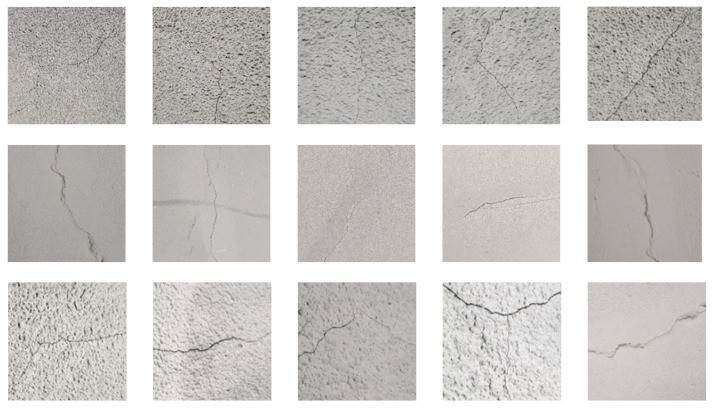
Tunnel crack example dataset.

Figure 9a displays the tunnel entrance scene, Figure 9b displays the image acquisition scene of the tunnel surface, while Figure 9c shows the tunnel crack images. The UAV collected 600 original tunnel crack images, which data augmentation techniques including random cropping, mirror flipping, brightness adjustment and image rotation expanded to over 1200 images. Python 3.9 was used to fuse the dataset collected by the UAV with publicly available datasets such as Road Crack dataset, Tunnel Crack and dataset CFD to obtain 3000 crack images as the dataset used in the crack segmentation experiments. And all the images are randomly divided into training set, validation set and test set according to the ratio of 70%, 20% and 10% using the script file. Part of the tunnel crack example dataset is shown in Figure 10.

Figure 9 and Figure 10 show the high authenticity and representativeness of the tunnel crack image dataset. The dataset was collected from operating tunnels and the images obtained after data augmentation such as flipping, brightness adjustment, etc. The dataset contains a wide range of crack patterns, such as fine irregular cracks, irregular cracks, and complex patterns caused by environmental factors. These reflect real-world challenges in tunnel crack detection. The dataset includes crack images under varying lighting conditions and shooting angles, which increase recognition difficulty and complexity. Uneven illumination, shadows and specular reflections within tunnels pose significant challenges for accurate crack identification. Varying shooting angles further complicate recognition by generating diverse crack appearances in images. The dataset also incorporates tunnel lining surface textures and background patterns that may mimic crack features, introducing interference to precise identification. Validation on this dataset confirms the proposed algorithm’s effectiveness and accuracy for detecting fine irregular tunnel cracks.

### 4.2. Experimental Environment and Evaluation Indicators

The experiments built the deep learning framework on CUDA 12.4, Python 3.9 and PyTorch 2.5.1, utilizing GPU for network training.

This paper assesses the model’s localization accuracy with precision (P), recall (Re), F1-score and mean average precision (mAP) to verify its accuracy and computational speed. It evaluates crack segmentation performance using Intersection over Union (IoU) and F1-score [35]. Precision is calculated as the ratio of correctly identified cracks to the total number of detected cracks. Recall measures the proportion of true crack samples that the model correctly identifies. The F1-score, which is the harmonic mean of precision and recall, provides a balanced measure of model performance. mAP calculates the mean average precision at an IoU threshold of 0.5. IoU measures the overlap between predicted and actual crack regions. The formulas are defined as

(14) $$\mathrm{Pr}=\frac{TP}{TP+FP},$$

(15) Re=TPTP+FN,

(16) F1=2×Pr×RePr+Re,

(17) $$mAP=\frac{1}{n}\sum_{i=1}^{n}\int_{0}^{1}P(R)dR,$$

(18) $$IoU=\frac{TP}{TP+FP+FN},$$

where *TP* denotes cracks correctly detected and classified by the network. *FN* represents the number of cracks that exist but remain undetected. *FP* indicates background regions misclassified as cracks [36,37]. The variable *n* corresponds to the number of target categories.

## 5. Experiment and Analysis

In order to verify the effectiveness of the proposed algorithm, we adopt a multi-source data fusion method, in which the self-acquired UAV dataset (600 images) is augmented with 1200 images by horizontal flipping, vertical flipping, rotational transformation, etc., and integrated with authoritative public datasets-tunnel Crack (800 tunnel crack images) and Crack Forest Datasets (CFD) (1000 complex-background crack images) to construct a comprehensive multi-scene dataset containing 3000 high-quality fracture images. Based on the 3000 crack-containing images selected from the captured images and the authoritative public dataset-tunnel, the dataset is cropped to a size of 512 × 512 pixels using the sliding window algorithm for model training, and the dataset is partitioned into the training, testing and validation sets according to 7:2:1.

### 5.1. Experimental Validation of Crack Localization Identification and Segmentation Algorithm Based on Improved YOLOX and UNETR++

The enhanced YOLOX algorithm examines collected image data with the goal of accurately identifying and locating fine irregular cracks in tunnels. It demonstrates strong detection capabilities, swiftly identifying crack regions and determining their precise locations. Subsequently, these images are processed by the UNETR++ model, which performs detailed segmentation. The model analyzes each pixel to differentiate between cracked and uncracked areas, thereby accurately defining the cracks’ shapes.

**Figure 11:**
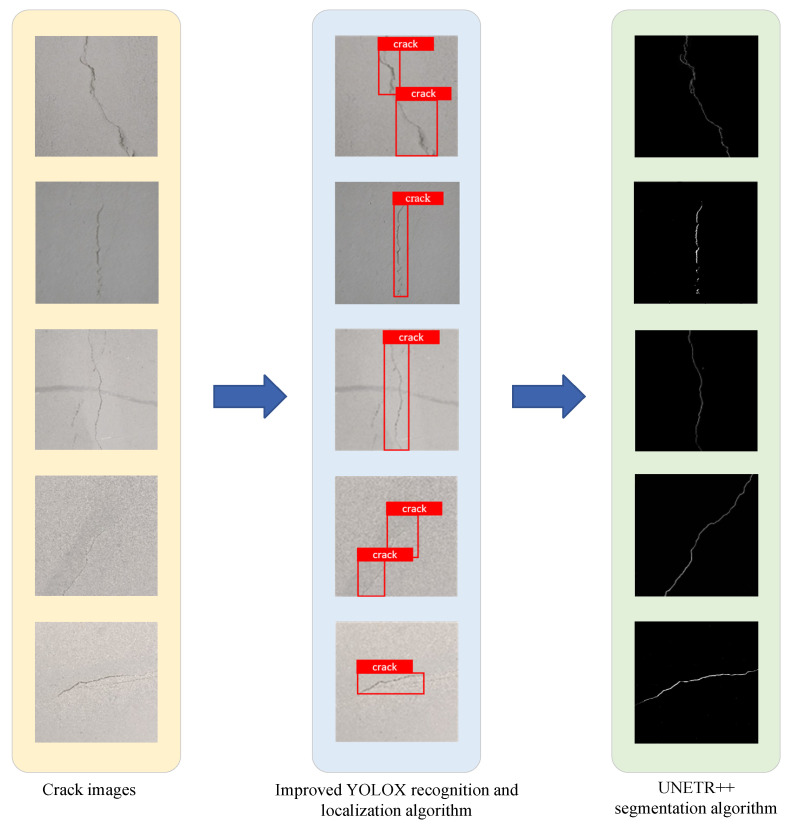
Fine irregular cracks detection and segmentation results.

Figure 11 illustrates the performance of the proposed algorithm across the tunnel crack recognition process. It efficiently identifies, localizes, and segments fine irregular cracks. The left panel shows the original crack image. The central panel displays the detection results from the improved YOLOX algorithm, which accurately pinpoints the cracks. The right panel presents the segmentation results from the UNETR++ algorithm, which precisely outlines the complete crack contours.

The experimental results show that our method performs well in recognizing fine irregular cracks. It works well even in tough situations like complicated backgrounds, a lot of noise and an uneven mix of positive and negative samples. This means that our improved YOLOX algorithm and UNETR++ segmentation are both strong and accurate for finding cracks in tunnels. The improved YOLOX algorithm cuts down on missed detections. It does this by making feature extraction better and strengthening the network’s structure. This makes it better at spotting tiny crack details. Also, the UNETR++ segmentation algorithm draws very accurate crack outlines. It does this by combining attention mechanisms with deep convolutional neural networks. This proves that our combined detection–localization–segmentation system is practical for tunnel crack problems.

### 5.2. Experimental Analysis of Improved YOLOX Crack Identification and Localization

#### 5.2.1. Ablation Experiment

The improved YOLOX algorithm proposed in this paper contains three key improvements, including the improvement of the EfficientNet backbone network, the introduction of the lightweighting module ECA and the improvement of the combined loss function. To systematically evaluate the individual contributions of these improvements, three ablation experiments were designed to analyze their respective impacts on model performance. All experiments were trained under identical conditions, including the same custom dataset, hyperparameter settings and computational environment. In this case, the hyperparameters were set to 200 training cycles, its input parameters were activated by the log-SoftMax function, the batch size was set to 4; the optimizer chosen was Adam, which set the initial learning rate set to 0.001; and the value of the model’s batch size was set to 8. The quantitative results are summarized in Table 2.

**Figure 12:**
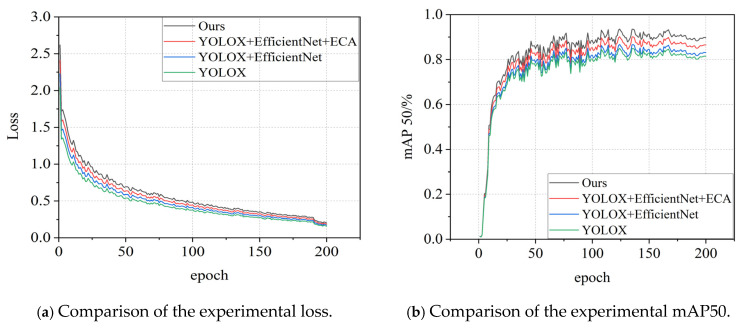
Comparative results of ablation experimental training.

The performance of the ablation experiment during training is demonstrated as shown in Figure 12, where the horizontal axis indicates the number of rounds (epochs) of training, and the loss convergence curves of the improved model compared with the Baseline model are shown in Figure 12a and the mAP50 curves are shown in Figure 12b.

As demonstrated in Figure 12a, the improved model exhibits superior convergence behavior, indicating its enhanced capability to comprehensively learn crack texture characteristics during training and stronger generalization performance. Figure 12b reveals that the average precision of the improved model stabilizes after 150 training epochs, consistently outperforming the baseline model by a significant margin. Furthermore, the absence of overfitting in the improved model demonstrates its improved accuracy and training stability.

A thorough analysis of the data presented in Table 2 and Figure 12 clearly reveals that the introduction of the EfficientNet architecture leads to a significant improvement in key evaluation metrics, with mAP50 increasing from 81.28% to 83.17%, and FPS rising from 47.65 to 55.49. This change intuitively demonstrates that the integration of EfficientNet not only significantly enhances the model’s recognition accuracy but also optimizes its processing speed, thereby improving operational efficiency. Furthermore, when the ECA mechanism is incorporated into the model already utilizing EfficientNet, the performance gains become even more pronounced: mAP50 jumps to 86.38%, and FPS slightly increases to 58.87. The ECA mechanism effectively boosts the weights of critical channels in the feature map, thereby enhancing the model’s ability to capture and leverage important features, further improving its recognition precision. The synergistic effect of the EfficientNet backbone and the ECA module not only enhances feature extraction and model training, but also accelerates inference speed through network structure optimization. This dual optimization strategy successfully achieves an ideal balance between model accuracy and efficiency, providing a more efficient and precise solution for practical applications.

#### 5.2.2. Comparison Experiment

To validate the performance of the proposed improved YOLOX algorithm in tunnel crack recognition, this paper conducts comparative experiments with four other models on the established dataset. The evaluation metrics of all algorithms are statistically analyzed, and Table 3 presents the comparative experimental results of recognition and localization algorithms.

From the results of the comparison experiments in Table 3, it can be seen that the proposed improved YOLOX algorithm shows significant advantages in several key indicators. In terms of detection precision, the F1-score of the improved YOLOX reaches 84.73%, which is somewhat improved compared with the original YOLOX, indicating that it performs better in the balance between precision and recall. Its precision (P) is 87.65%, which is leading among the compared algorithms, implying that the algorithm has a lower false detection rate for the positive class samples; the recall (R) is 82.27%, which also shows a better performance. Meanwhile, mAP50 reaches 89.14%, outperforming Faster R-CNN, YOLOv3, YOLOv5, YOLOX YOLOv8, and Mobile-Former by 12.09%, 14.04%, 10.90%, 9.67%, 5.43% and 5.97%, respectively, which is an excellent performance in terms of the comprehensive performance of target localization and classification. In terms of speed, Improved—YOLOX achieves an FPS of 62.28, which is slightly higher than Mobile—Former and significantly better than the YOLO series of algorithms, demonstrating the efficiency of real-time processing. Although FLOPs/G is slightly higher compared to Mobile-Former, Improved YOLOX has a better balance of accuracy and speed. Therefore, by optimizing the model structure and computational process, improved YOLOX significantly improves the detection accuracy while maintaining the efficient inference speed, which is especially suitable for scenarios requiring both accuracy and real-time performance, and provides a more reliable and efficient solution for object detection tasks.

To further validate the superior performance of the improved YOLOX model in detecting fine and irregular texture cracks in tunnels, we selected four different object detection models for comparative analysis. To objectively evaluate the performance of each model, we conducted recognition tests using a set of tunnel lining crack images.

**Figure 13:**
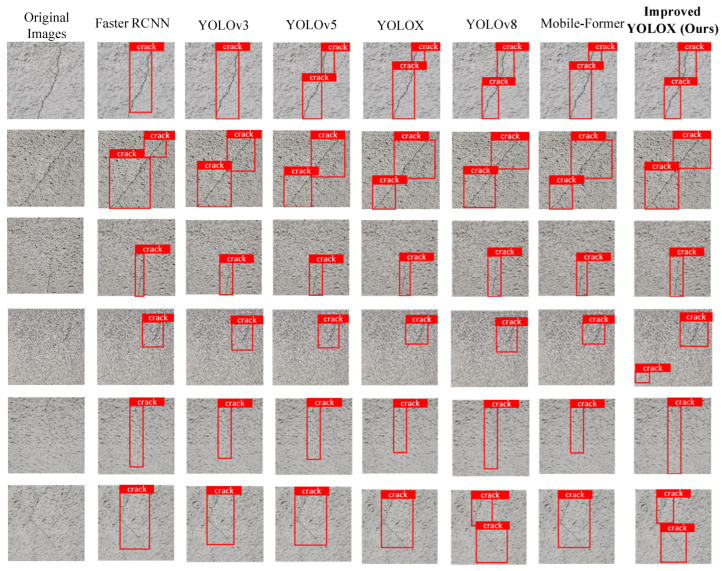
Comparison results of different recognition algorithms for cracks.

The validation process specifically focused on irregular crack textures and fine texture features, as these features are critical for assessing crack severity and determining appropriate maintenance measures. Figure 13 presents the recognition test results, with the charts visually demonstrating performance differences among models in detecting fine and irregular texture cracks.

Figure 13 demonstrates a comprehensive comparative analysis of model performance in detecting fine irregular texture cracks in tunnel linings, covering critical aspects including crack localization accuracy, morphological completeness and texture detail clarity. The results show that the improved YOLOX model exhibits superior performance across all these metrics. The model achieves precise crack localization with minimal positional errors, demonstrating its powerful feature extraction capability and refined network architecture. Moreover, it maintains high recognition accuracy even in complex backgrounds. Figure 13 further validates the model’s outstanding performance in fine texture crack detection, showing more accurate localization while preserving crack integrity and continuity. This confirms the improved YOLOX algorithm’s higher detection precision for tunnel lining cracks, making it more suitable for engineering applications.

### 5.3. Experimental Analysis of UNETR++ Segmentation

**Figure 14:**
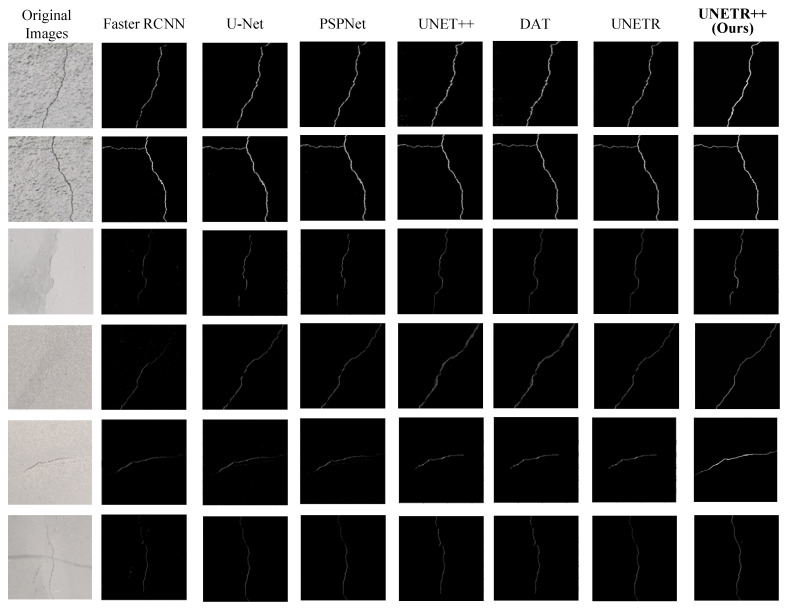
Comparison results of different segmentation algorithms for cracks.

To extract crack information from images, the improved YOLOX network feeds identified crack images into the UNet++ network, achieving automated crack extraction. To validate the effectiveness of the UNETR++-based crack segmentation algorithm, we compare it with mainstream semantic segmentation algorithms: Faster R-CNN, U-Net, UNet++ and UNETR. The comparative segmentation results are shown in Figure 14.

The visualization results demonstrate that UNETR++ achieves higher overall segmentation accuracy with fewer misidentifications and clearer boundary delineation for fine irregular tunnel cracks compared to baseline methods. Conventional Faster R-CNN fails to accurately capture crack morphology due to typically fine and irregular crack patterns. Although U-Net improves the segmentation accuracy under low noise conditions, it still has difficulty in accurately delineating the cracks, and PSPNet has a relatively low accuracy in recognizing the cracks, and the morphology of some cracks is not accurately captured, especially in the region where the cracks are fine and irregular, and the segmentation results have a large deviation from the real cracks in the original image. Enhanced versions of U-Net, UNet++ and UNETR achieve better crack segmentation than the original U-Net, but exhibit limited performance for fine irregular textures, prone to missed or false detections. In addition, DAT has leakage detection for some fine irregular cracks, and compared with UNETR++, DAT needs to improve its stability and accuracy when dealing with tunnel crack segmentation under complex background and noise interference. UNETR++ shows superior performance in tunnel crack segmentation, maintaining accuracy under complex backgrounds and noise while preventing misclassification and missed detections. This capability makes UNETR++ excel in practical applications, handling fine-texture cracks and complex backgrounds without compromising precision.

**Figure 15:**
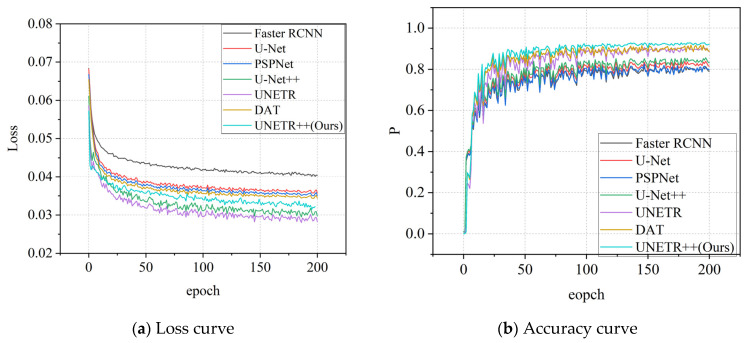
Training curve chart.

Figure 15 presents the training performance comparison between UNETR++ and mainstream semantic segmentation algorithms (Faster R-CNN, U-Net, PSPNet, UNet++, DAT and UNETR). The x-axis represents training epochs, with Figure 15a displaying the loss convergence curves and Figure 15b showing the accuracy curves.

Figure 15a clearly demonstrates that the proposed UNETR++ model achieves superior convergence performance compared to other segmentation algorithms. This indicates UNETR++’s enhanced capability to comprehensively capture complex crack texture characteristics during training. Its optimized network architecture and algorithms enable more efficient extraction of critical features from training data, consequently exhibiting stronger generalization capacity for accurate crack feature identification across diverse scenarios. Figure 15b further reveals UNETR++’s outstanding performance in crack segmentation, particularly in accuracy metrics, achieving more precise crack region delineation that provides more reliable support for subsequent crack detection and analysis tasks.

Table 4 compares the performance of UNETR++ with mainstream segmentation models on the tunnel crack dataset.

Table 4 shows the results of comparative experiments of different segmentation algorithms on a range of performance metrics. As can be seen from the table, UNETR++ excels in several key performance metrics. Specifically, UNETR++ achieves 85.3% in Precision (P/%) and 96.1% in Recall (R/%), both of which are higher than other algorithms. In addition, UNETR++ also achieved 79.8% in IoU. On F1-score/%, UNETR++ leads with 90.2%, showing a good balance between precision and recall. In terms of computational efficiency, UNETR++ has an FPS of 45.3, which is not the highest, but the algorithm proposed in this paper is still competitive considering its excellent performance in other performance metrics. In addition, UNETR++ has a FLOPs/G of 41.7 and a parameter number of 12.3M, which indicates that the model has good computational efficiency and model complexity control while maintaining high performance. Overall, the UNETR++ algorithm provides a reliable and efficient solution in segmentation tasks, proving its potential for application in image segmentation through significant improvements in key performance metrics such as precision, recall, F1-score, and IoU.

## 6. Conclusions

According to the characteristics of tunnel crack images, an improved YOLOX recognition and localization algorithm is used to identify and locate fine and irregular crack images, and the UNETR++ algorithm is used to precisely segment the detected crack images. The improved model incorporates the EfficientNet–ECA module to enhance the efficiency and accuracy of small target detection. In the tunnel crack dataset test, the four indices of P, R, IoU and F1 achieved excellent results. Although the model is not optimal in terms of computational efficiency, the algorithm proposed in this paper is able to meet the re-requirements of crack segmentation on detection accuracy, and effectively improves the efficiency and accuracy of tunnel crack segmentation. The research results in this paper provide reliable data support for subsequent fracture risk assessment. In the future, we will further explore the accurate identification of cracks under complex environmental conditions.

## Figures and Tables

**Table 1:** EfficientNet baseline network.

Stage	Operator	Resolution	Channels	Layers
1	Conv 3 × 3	224 × 224	32	1
2	MBConv 1, k3 × 3	112 × 112	16	1
3	MBConv 6, k3 × 3	112 × 112	24	2
4	MBConv 6, k5 × 5	56 × 56	40	2
5	MBConv 6, k3 × 3	28 × 28	80	3
6	MBConv 6, k5 × 5	14 × 14	112	3
7	MBConv 6, k5 × 5	14 × 14	192	4
8	MBConv 6, k3 × 3	7 × 7	320	1
9	Conv 1 × 1&Pooling&FC	7 × 7	1280	1

**Table 2:** Comparison of the identification accuracy of ablation experiments.

Method	mAP 50/%	FPS
YOLOX	81.28	47.65
YOLOX + EfficientNet	83.17	55.49
YOLOX + EfficientNet + ECA	86.38	58.87
Improved YOLOX (Ours)	89.14	62.28

**Table 3:** The comparative experimental results of recognition and localization algorithms.

Algorithm	F1-Score/%	P/%	R/%	mAP50/%	FPS	FLOPs/G	Parameter/M
Faster RCNN	80.21	82.49	78.27	79.52	18.62	138.73	41.52
YOLOv3	78.2	80.83	74.56	78.17	40.38	65.21	61.23
YOLOv5	79.34	81.31	75.39	80.38	42.65	98.73	7.29
YOLOX	82.31	84.28	78.31	81.28	47.65	25.69	9.03
YOLOv8	85.18	86.53	83.72	85.92	58.24	26.85	10.52
Mobile-Former	83.53	84.68	82.19	83.71	61.53	24.16	6.82
Improved YOLOX (ours)	84.73	87.65	82.27	89.14	62.28	26.42	8.75

**Table 4:** Comparative experiments on segmentation algorithms.

Algorithm	P/%	R/%	IoU/%	F1-Score/%	FPS	FLOPs/G	Parameter/M
Faster RCNN	58.0	87.0	52.7	70.1	12.3	200.3	41.2
U-Net	72.5	90.3	66.1	80.3	25.2	152.3	34.5
PSPNet	74.5	89.5	66.7	80.8	20.3	183.6	46.7
UNet++	77.2	90.4	70.4	83.6	31.8	172.4	27.4
DAT	78.6	90.8	70.2	83.9	27.9	167.2	38.6
UNETR	80.7	93.2	74.3	86.1	36.3	62.3	18.9
UNETR++(Ours)	85.3	96.1	79.8	90.2	45.3	41.7	12.3

## Data Availability

The data that support the findings of this study are available from the corresponding author upon reasonable request.
